# Toward ED Based Cardio Oncology Pathways From a Nationwide Arrhythmia Cohort

**DOI:** 10.1002/joa3.70167

**Published:** 2025-08-06

**Authors:** Yalcin Golcuk

**Affiliations:** ^1^ Department of Emergency Medicine Faculty of Medicine, Muğla Sıtkı Koçman University Muğla Turkey

**Keywords:** atrial fibrillation, cancer‐related arrhythmias, cardio‐oncology, emergency medicine, sepsis‐induced tachyarrhythmia

To the Editor,

The landmark study by Kobayashi and Kusano [1] provides invaluable insights into arrhythmia patterns among cancer patients using Japan's nationwide JROAD‐DPC database. Despite the rising incidence of cardiovascular complications in oncology patients, few large‐scale studies have examined arrhythmia profiles across cancer subtypes and treatment trajectories in real‐world emergency settings. As emergency physicians (EPs) managing acute cardio‐oncologic complications, we commend this study and wish to highlight three findings with critical implications for emergency care, along with opportunities to strengthen clinical translation.

The significantly higher rate of emergency admissions among cancer patients with arrhythmias (58.9% vs. 57.6% in non‐cancer patients; *p* < 0.05) underscores a growing challenge for emergency departments (EDs). This finding aligns with global trends showing increasing cardiovascular emergencies related to malignancy due to aging populations and the widespread use of cardiotoxic therapies [2]. However, the underlying factors contributing to these admissions—whether related to arrhythmia severity, cancer progression, or gaps in outpatient care—remain insufficiently explored. Clarifying these drivers could enable the implementation of targeted interventions such as rapid‐access cardio‐oncology clinics or ED‐based clinical triggers, including recurrent arrhythmia or recent chemotherapy exposure, for early cardiology consultation.

The predominance of atrial fibrillation or flutter (AF/AFL) among cancer‐associated arrhythmias, comprising 70.6% of cases, has direct implications for acute management in the ED [3]. While the authors report lower anticoagulant use among cancer patients, EPs frequently face complex therapeutic dilemmas. Rhythm control may be constrained by QT‐prolonging chemotherapies, and anticoagulation decisions must be cautiously balanced against risks of bleeding, especially in the context of thrombocytopenia or mucosal tumors. ED‐specific algorithms would benefit from integrating oncology‐informed variables such as current drug profiles, platelet counts, and malignancy characteristics to individualize AF management.

The observed frequencies of pneumonia (7.41%) and sepsis (2.26%) as clinical triggers for arrhythmia underscore the role of systemic inflammation in acute cardiac dysrhythmias. In the ED, febrile presentations in oncology patients often signal infection‐related arrhythmogenic potential [4]. Embedding arrhythmia screening protocols, such as mandatory electrocardiograms for patients with febrile neutropenia, within sepsis bundles may support early detection of tachyarrhythmias and timely antimicrobial or hemodynamic intervention.

This study's use of nationwide claims data effectively captures broad epidemiologic trends; although coding limitations restrict the granularity of arrhythmia etiology and disease trajectory. Future prospective studies that correlate arrhythmia subtypes with cancer stage, treatment modality, and ED‐centered outcomes such as return visits or time to cardiology consultation may yield actionable risk stratification models. Moreover, the absence of disposition‐level data represents a missed opportunity to inform quality improvement efforts in emergency cardio‐oncology workflows.

In conclusion, Kobayashi and Kusano's study compellingly validates the evolving role of the ED in cardio‐oncology care. To operationalize these insights, we propose collaborative development of three core strategies: ED‐specific risk scores for arrhythmias in cancer patients, standardized AF management pathways for patients with thrombocytopenia, and integrated infection‐arrhythmia screening tools. We believe this is a critical juncture to embed cardio‐oncology into ED frameworks, advancing from reactive stabilization toward anticipatory, coordinated care.

## Author Contributions

**Yalcin Golcuk:** conceptualization, writing – original draft, writing – review and editing.

## Conflicts of Interest

The author declares no conflicts of interest.

## Linked Articles

This article is linked to Kobayashi and Kusano's article. To view this article, visit https://doi.org/10.1002/joa3.70079.
